# Estrogen Receptor β Participates in Alternariol-Induced Oxidative Stress in Normal Prostate Epithelial Cells

**DOI:** 10.3390/toxins13110766

**Published:** 2021-10-29

**Authors:** Karolina Kowalska, Marta Justyna Kozieł, Kinga Anna Urbanek, Dominika Ewa Habrowska-Górczyńska, Kamila Domińska, Agnieszka Wanda Piastowska-Ciesielska

**Affiliations:** 1 Department of Cell Cultures and Genomic Analysis, Medical University of Lodz, Zeligowskiego 7/9, 90-752 Lodz, Poland; marta.koziel@umed.lodz.pl (M.J.K.); kinga.urbanek@umed.lodz.pl (K.A.U.); dominika.habrowska@umed.lodz.pl (D.E.H.-G.); agnieszka.piastowska@umed.lodz.pl (A.W.P.-C.); 2 Department of Comparative Endocrinology, Medical University of Lodz, Zeligowskiego 7/9, 90-752 Lodz, Poland; kamila.dominska@umed.lodz.pl

**Keywords:** alternariol, mycotoxin, prostate, oxidative stress, DNA damage

## Abstract

*Alternaria* toxins are considered as emerging mycotoxins, however their toxicity has not been fully evaluated in humans. Alternariol (AOH), the most prevalent *Alternaria* mycotoxin, was previously reported to be genotoxic and to affect hormonal balance in cells; however, its direct molecular mechanism is not known. The imbalance in androgen/estrogen ratio as well as chronic inflammation are postulated as factors in prostate diseases. The environmental agents affecting the hormonal balance might participate in prostate carcinogenesis. Thus, this study evaluated the effect of two doses of AOH on prostate epithelial cells. We observed that AOH in a dose of 10 µM induces oxidative stress, DNA damage and cell cycle arrest and that this effect is partially mediated by estrogen receptor β (ERβ) whereas the lower tested dose of AOH (0.1 µM) induces only oxidative stress in cells. The modulation of nuclear erythroid-related factor 2 (Nrf2) was observed in response to the higher dose of AOH. The use of selective estrogen receptor β (ERβ) inhibitor PHTPP revealed that AOH-induced oxidative stress in both tested doses is partially dependent on activation of ERβ, but lack of its activation did not protect cells against AOH-induced ROS production or DNA-damaging effect in case of higher dose of AOH (10 µM). Taken together, this is the first study reporting that AOH might affect basic processes in normal prostate epithelial cells associated with benign and malignant changes in prostate tissue.

## 1. Introduction

Mycotoxins, as the toxic metabolites of fungi, are present in every day human diets, both in processed as well as unprocessed food. Chronic exposure to mycotoxins represents a global health issue and in recent years a number of studies have been conducted to evaluate and shed more light on how most common mycotoxins might affect human and animal health [1].

Alternariol (AOH) is one of the most abundant mycotoxins produced by *Alternaria* spp.—a commonly found black mold, affecting food products and buildings [2]. AOH is considered an emerging mycotoxin with safety exposure limits not established yet by the European Food Safety Authority (EFSA) due to insufficient research carried out so far [2]. Nevertheless, AOH is reported to act as an inhibitor of topoisomerase—an enzyme crucial to the proliferation of cells [3]; it also induces genotoxicity and oxidative stress in cells [4], affects cell cycle and apoptosis in in vitro cell models [5,6]. Besides genotoxicity as a main concern, AOH in a dose of 10 µM was also reported to modulate the immune response in cells [7]. In addition, it acts as an endocrine disruptor via modulation of androgen receptor (AR) signaling with the half maximal effective concentration (EC50) of 269.4 μM [8]. AOH was also reported to possess estrogenic activity similar to phytoestrogen daidzein or the environmental estrogen bisphenol A (BPA) observed in cell-free system with EC50 of 3.1 μM ± 2.9 μM [9]. The study also showed that AOH possess higher binding affinity to ERβ rather than ERα [9]. However, data are not consistent though—a mixture of different *Alternaria* mycotoxins was reported to trigger an anti-estrogenic rather than estrogenic in endometrial cancer cell line Ishikawa treated with AOH (0.1–50 µg/mL) and 1 nM estradiol (E2) [10]. Thus, it is highly possible that the estrogenic effect of AOH might be cell type- dependent and still it has not been evaluated in prostate epithelial cells.

Oxidative stress is defined as a state of imbalance between production of high energy molecules called reactive oxygen species (ROS), and the respective detoxifying mechanisms [11]. An increased amount of ROS in cells might be associated with activation of various transcription factors including nuclear factor kappa-light-chain-enhancer of activated B cells (NF-κB), nuclear factor erythroid 2-related factor 2 (Nrf2), tumor suppressor p53 and hypoxia inducible factor 1 α (HIF-1α) [11]; it can also induce DNA damage, disturbances in cell cycle progression and modulation of gene expression. Oxidative stress participates in the two most common prostate diseases: benign prostate hyperplasia (BPH) and prostate cancer (PCa). Both diseases are hormone- and age- related [12]. Modulation of the estrogen and androgen signaling pathway participates in prostate cells proliferation and growth [13]. The androgen/estrogen ratio in prostate tissue is essential, but the role of ERβ remains questionable. On one hand knockout of ERβ in mice resulted in decreased proliferation of prostate cells, yet on the other hand, in a prostate cohort study, ERβ expression was correlated with decreased survival of patients [14]. This fact might be partially associated with different isoforms of ERβ, which seem to play contradictory roles in prostate tissue.

Nevertheless, environmental agents which might affect testosterone/estrogen ratio in prostate tissue may also participate in inflammation or carcinogenesis in the prostate. As mentioned before, AOH was reported to both affect AR and ERβ [8,15], and it is possible that the linking molecular pathway associated with these effects is induction of oxidative stress in cells. Thus, the aim of the study was firstly to evaluate the effect of AOH on the induction of oxidative stress in normal prostate cell line PNT1A and secondly to check if it is associated with the activation of ERβ. We observed that AOH induces oxidative stress, DNA damage, and cell cycle arrest in G2/M cell cycle phase in normal prostate epithelial cells. Moreover, AOH affects prostate cells migration, metalloproteinases activity and triggers immune response.

## 2. Results

### 2.1. AOH Significantly Affects Viability of Prostate Epithelial Cells

**Figure 1:**
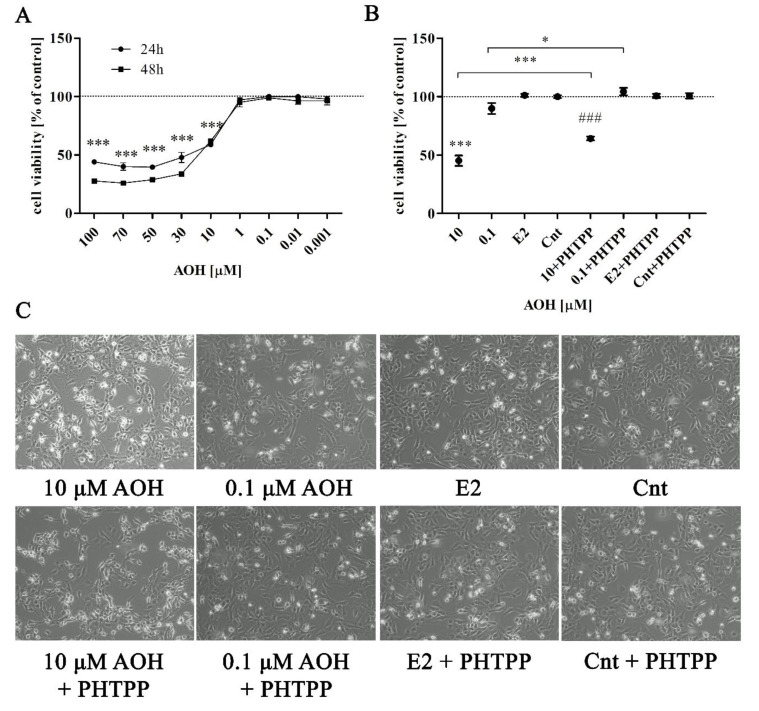
AOH affects viability of prostate epithelial cells. (**A**) The dose-dependent viability curve obtained after 24 and 48 h. (**B**) Cells viability results after 24 h. The viability was evaluated with Alamar Blue^®^ assay. The results are expressed as mean ± SE. *p* value lower than 0.05 was considered as statistically significant. One-way ANOVA was used for statistical analysis. (**C**) Morphological changes in cells observed after 24 h in optical microscope, magnitude 100×. * *p* < 0.05, *** *p* < 0.001 as compared to control, ### *p* < 0.001 as compared to Cnt PHTPP. AOH—alternariol, PHTPP—ERβ inhibitor, Cnt—control. The cell viability assay was carried out in 6 replications.

Firstly, the effect of AOH on PNT1A cell was evaluated in a dose- and time- dependent manner (Figure 1A). It was observed that AOH in a dose range between 10 and 100 µM significantly influenced the viability of prostate epithelial cells (*** *p* < 0.001), while concentrations below 10 µM caused no effect, both after 24 and 48 h. Based on these results, the lowest dose of AOH for which the decrease in cell viability (10 µM) and a dose for which no decrease was observed were chosen for the rest of experiment as a higher dose (10 µM) and a lower dose (0.1 µM) of AOH, respectively. Then, the cell viability was once again evaluated to verify if ERβ is involved in the viability reduction caused by AOH (Figure 1B). Treatment of PNT1A cells with 10 µM AOH and ERβ inhibitor PHTPP also caused decrease in cell viability as compared to cells treated only with PHTPP, however the decrease was significantly smaller compared to AOH treatment alone (*** *p* < 0.001). A similar effect was observed for higher dose of AOH (* *p* < 0.05). Estradiol (E2) in physiological concentration has no effect on PNT1A cell viability. The observed changes in cell viability were also reflected in cell morphology (Figure 1C)—cells treated with higher dose of AOH showed increased visibility of nuclei and contracted cell shape.

### 2.2. AOH Induces Oxidative Stress in PNT1A Cells

**Figure 2:**
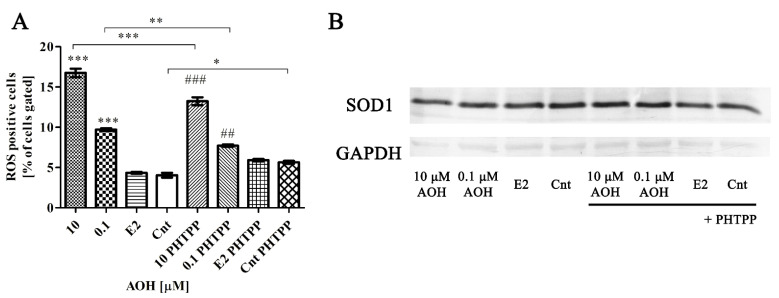
Blocking of ERβ decreases AOH- induced oxidative stress in PNT1A cells. (**A**) ROS positive cells counted with Muse^®^ Cell Analyzer. (**B**) Representative results of Western blot analysis of SOD1 expression. All results are expressed as mean ± SE. One-way ANOVA was used for statistical analysis. *p* < 0.05 was considered as statistically significant, * *p* < 0.05, ** *p* < 0.01, *** *p* < 0.001, ## *p* < 0.01, ### *p* < 0.001 as compared to Cnt PHTPP. AOH—alternariol, PHTPP—ERβ inhibitor, Cnt—control. The experiments were run in 3 replications.

In the next part of experiment we evaluated if the decreased viability of PNT1A cells is associated with oxidative stress and DNA damage. Firstly, we observed, as assumed, that AOH induces oxidative stress in PNT1A cells in a dose-dependent manner (Figure 2A). Similarly to the changes in the viability results, blocking of ERβ with PHTPP caused decrease in generation of ROS as compared to cell treated only with AOH for both doses used in the experiment (*** *p* < 0.001 and ** *p* < 0.01, respectively for 10 µM and 0.1 µM AOH). However, inhibition of ERβ was not sufficient to protects cells against AOH induced generation of ROS. The induction of oxidative stress was also associated with the modulation of superoxide dismutase 1 (SOD1) expression on gene and protein level (Figure 2B). An increased expression of SOD-1 was observed after treatment with 10 µM AOH as compared to control, whereas a contradictory effect was observed for treatment with AOH + PHTPP as compared to Cnt + PHTPP. For the lower dose a similar but smaller effect was observed (Table 1).

We also observed that induction of oxidative stress was associated with modulation of the expression of *SOD1*, *HIF-1α* and *RelA* (Table 2). In case of all tested genes the changes observed after AOH treatment were not significant, however the significant changes in the expression of *HIF-1α* (** *p* < 0.01) and *RelA* were observed after addition of PHTPP to AOH-treated cells as compared to AOH treatment alone (*** *p* < 0.001). On the protein level we observed an almost 60% increase in *SOD1* expression after treatment with higher dose of AOH and 30% increase in the case of lower AOH dose compared to control cells (Figure 2B). Addition of PHTPP reduced the expression to 87% for higher dose and 94% of untreated cells. The expression of *SOD1* in cells treated only with PHTPP was, similar to the RT-qPCR results, slightly increased (22%) compared to control cells.

### 2.3. AOH Induces DNA Damage in PNT1A Cells

**Figure 3:**
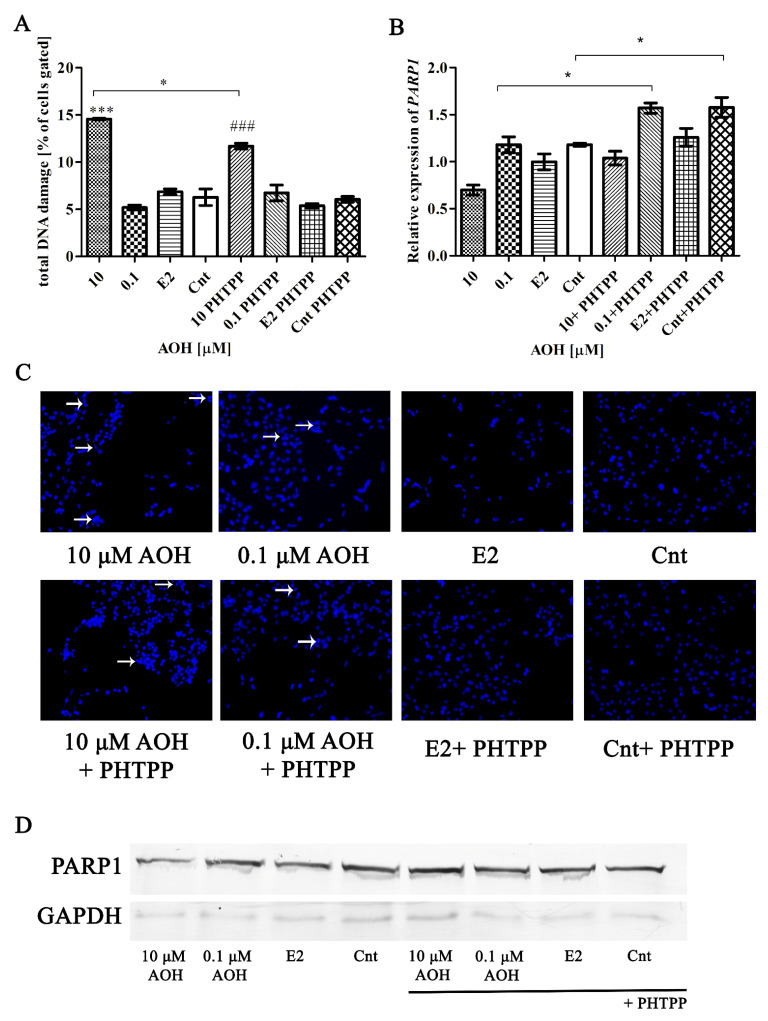
AOH induces DNA damage in PNT1A cells. (**A**) DNA damage was counted on Muse^®^ Cell Analyzer and expressed as % of cells gated. (**B**) Relative expression of *PARP1* obtained in RT-qPCR. The results are expressed as mean ± SE. (**C**) DAPI staining of the nuclei of cells. White arrows point at the cells with changed of fragmented nuclei. (**D**) Representative results of Western blot analysis of the expression of cleaved-PARP1 protein. One way ANOVA was used for statistical analysis. *p* < 0.05 was considered as statistically significant. * *p* < 0.05, *** *p* < 0.001 as compared to Cnt, ### *p* < 0.001 as compared to Cnt PHTPP. AOH—alternariol, PHTPP—ERβ inhibitor, Cnt—control. The experiments were run in 3 replications.

Next, we evaluated if ERβ participates in AOH-induced DNA damage in cells. Firstly, it was observed that higher concentration of AOH, as expected, induced DNA damage in PNT1A cells (Figure 3A). Addition of PHTPP, similarly to oxidative stress results, significantly decreased the induction of DNA damage as compared to AOH treatment alone (* *p* < 0.01), but was not sufficient to completely prevent it (### *p* < 0.01 as compared to Cnt + PHTPP). The DNA damage was also visible in DNA staining as fragmented and not round-shaped nuclei of cells (Figure 3C). We also evaluated the expression of poly [ADP-ribose] polymerase 1(PARP1) both on mRNA and protein level, due to the fact that it acts as a first response to DNA damage in cells. We observed that a higher dose of AOH decreased expression of *PARP1* (Figure 3B). In case of both doses of AOH, blocking of ERβ caused increase in the expression of *PARP1*, statistically significant for lower dose of AOH (* *p* < 0.05). The increase in the expression was also present in control cells treated only with PHTPP as compared to control (* *p* < 0.05). On the protein level, decreased expression of cleaved PARP1 was found in cells treated with a higher dose of AOH as compared to non-treated cells (Table 3). Cells treated with PHTPP presented similar expression of cleaved PARP1 in all tested doses.

### 2.4. AOH Induces Cell Cycle Arrest in G2/M Cell Cycle Phase

**Figure 4:**
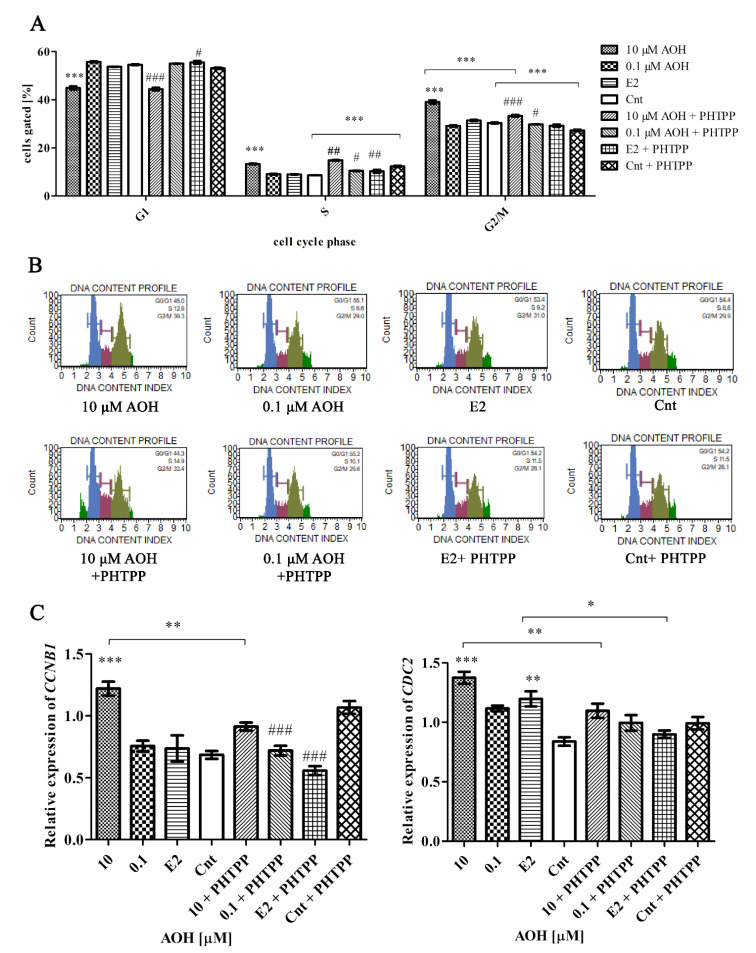
AOH induces cell cycle arrest in G2/M cell cycle phase. (**A**) The results of cell cycle analysis conducted with Muse^®^ Cell Analyzer. The results are expressed as a mean ± SE of the % of cells gated. (**B**) Representative results of the flow cytometry of cell cycle analysis. (**C**) RT-qPCR results of the relative expression of *CCNB1* and *CDC2*. The results are expressed as mean ± SE. One way ANOVA was used for statistical analysis. *p* < 0.05 was considered as statistically significant. * *p* < 0.05, ** *p* < 0.01, *** *p* < 0.001 as compared to Cnt, # *p* <0.05, ## *p*< 0.01, ### *p* < 0.001 as compared to Cnt PHTPP. AOH—alternariol, PHTPP—ERβ inhibitor, Cnt—control, CCNB1—cyclin B1, CDC2—cyclin-dependent kinase 1. The experiments were run in 3 replications.

The modulation of oxidative stress might be also associated with modulation of the progression of cell cycle. Our previous studies showed that mycotoxins modulate the progression of G2/M cell cycle phase [16,17]. Similarly to other estrogenic mycotoxin zearalenone, AOH in higher dose caused significant decrease in G1 cell cycle phase with simultaneous increase in S and G2/M cell cycle phase indicating cell cycle arrest in G2/M cell cycle phase (Figure 4A,B). In case of G2/M cell cycle phase, the addition of PHTPP caused statistically significant decrease in the number of cells in G2/M cell cycle phase (*** *p* < 0.05), however addition of PHTPP was not sufficient to counteract an increase in the number of PNT1A cells in G2/M phase. Similar effect was observed for control and control + PHTPP cells (*** *p* < 0.05). The progression of G2/M cell cycle phase is regulated by cyclin B1 (*CCNB1*) and cyclin-dependent kinase 1 (*CDC2*). We evaluated the expression of *CCNB1* and *CDC2* and observed that AOH in the dose of 10 µM significantly induces expression of both genes (*** *p* < 0.001) as compared to control cells (Figure 4C). No significant increase was observed for lower dose of AOH. For both genes, the addition of PHTPP to a higher dose of AOH caused decreased expression as compared to AOH treatment alone (** *p* < 0.01). Similar effect was observed for estrogenic control in the expression of CDC2 (* *p* < 0.05).

### 2.5. AOH Modulates Nrf2 Signaling Pathway

**Figure 5:**
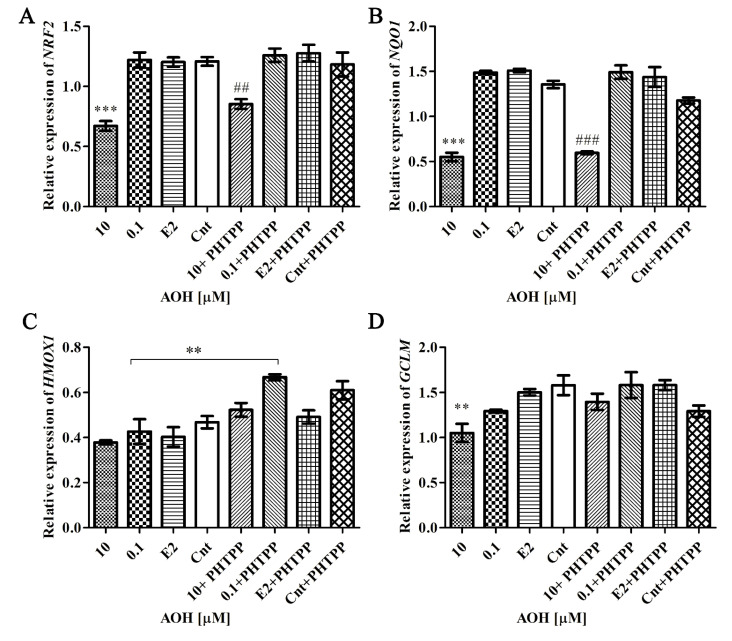
Relative expression of Nrf2 signaling pathway components. The relative expression of *NRF2* (**A**), *NQO1* (**B**), *HMOX1* (**C**), and *GCLM* (**D**) obtained in RT-qPCR and expressed as mean± SE. *H3F3A*, *RPLP0* and *RPS17* were used as house-keeping genes. One- way ANOVA was used for statistical analysis. *p* < 0.05 was considered as significant. ** *p* < 0.01, *** *p* < 0.001 as compared to control, ## *p* < 0.01, ### *p* < 0.001 as compared to Cnt + PHTPP. The experiment was run in 3 replications. AOH—alternariol, PHTPP—ERβ inhibitor, Cnt—control, Nrf2—nuclear factor erythroid 2-related factor 2, NQO1—NAD(P)H dehydrogenase [quinone] 1, HMOX—heme oxygenase 1, GCLM—glutamate-cysteine ligase regulatory subunit, *RPS17*—Ribosomal protein S17, RPLP0—ribosomal protein P0, *H3F3A*—histone H3.3A.

The response to oxidative stress is mainly associated with activation of detoxifying enzymes and Nrf2 signaling pathway. Thus, we evaluated if AOH-induced oxidative stress is associated with activation of Nrf2 signaling pathway (Figure 5). We observed that *NRF2* and its responsive element NAD(P)H quinone dehydrogenase 1 (*NQO1*) was significantly decreased after exposure to 10 µM AOH as compared to control (*** *p* < 0.001). In the case of other responsive element glutamate-cysteine ligase regulatory subunits (*GCLM*), a similar effect was observed but was not statistically significant. Interestingly, we did not observe any change in heme oxygenase 1 (*HMOX*), indicating that it does not participate in the AOH-induced oxidative stress response. Although a slight increase in the expression of *NRF2*, *NQO1,* and *GCLM* was observed after addition of PHTPP, the changes were not significant.

### 2.6. AOH Modulates Motility of Prostate Epithelial Cells

**Figure 6:**
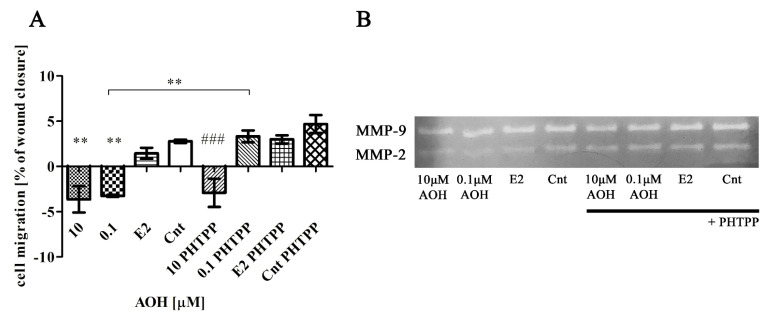
AOH decreases migration of PNT1A cells. (**A**) Migration of cells was evaluated with scratch assay. (**B**) Zymography was used to evaluate the activity of MMP-9 and MMP-2. The results are expressed as mean ± SE. One- way ANOVA was used for statistical analysis. *p* < 0.05 was considered as statistically significant. ** *p* < 0.01 as compared to Cnt, ### *p* < 0.001 as compared to Cnt PHTPP. AOH—alternariol, PHTPP—ERβ inhibitor, Cnt—control. The experiments were run in 3 replications.

The motility of cells is crucial to maintain a proper functioning of cells. Thus, in the next step we evaluated migration of cells after treatment with AOH (Figure 6A). In both tested doses of AOH we observed a significant (** *p* < 0.01) decrease in cell migration as compared to control cells. The blocking of ERβ (+PHTPP) lowered the effect of AOH in both tested doses; however, it was significant only for the lower dose of AOH (** *p* < 0.01). The lower migration of cells observed after AOH treatment might be associated with decreased activity of metalloproteinases (MMP-2 and MMP-9), detected during zymography assay (Table 4, Figure 6B). The higher decrease in MMP-2 activity was observed for 0.1 µM AOH and addition of PHTPP increased that. No such effect was observed for 10 µM AOH. In case of MMP-9 activity AOH decreased it slightly in both doses and similarly to MMP-2 activity, addition of PHTPP increased it, although the effect for lower dose of AOH was more detectable.

### 2.7. AOH Affects Inflammatory Response in PNT1A Cells

**Figure 7:**
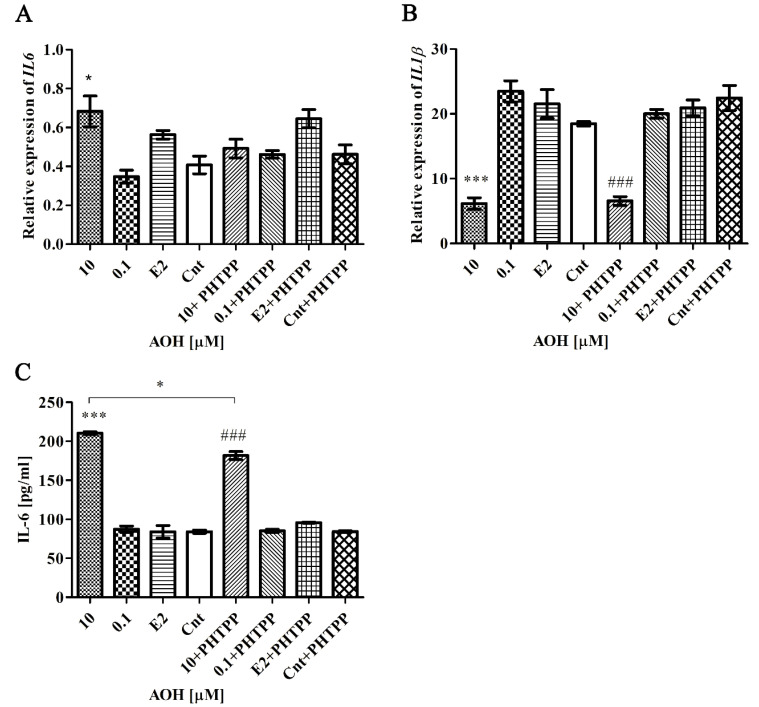
The relative expression of *IL6* and *IL-1β*. (**A**,**B**) The relative expression of *IL6* and *IL-1β* obtained in RT-qPCR and expressed as mean ± SE. *H3F3A*, *RPLP0* and *RPS17* were used as house-keeping genes. One way ANOVA was used for statistical analysis. *p* < 0.05 was considered as significant. (**C**) The relative expression of IL6 obtained in ELISA test. The results are expressed as mean ± SE of two replications. * *p* < 0.05, *** *p* < 0.001 as compared to control, ### *p* < 0.001 as compared to Cnt + PHTPP. AOH—alternariol, PHTPP—ERβ inhibitor, Cnt—control, IL6—interleukin 6, IL-1β—interleukin 1 subunit β, *RPS17*—Ribosomal protein S17, RPLP0—ribosomal protein P0, *H3F3A*—histone H3.3A.

It was previously suggested that AOH affects the inflammatory response in cells [18]. We observed that 10 µM AOH significantly affect expression of interleukin 6 (IL-6), both on mRNA and protein level. The expression of *IL6* was significantly increased after 10 µM AOH (* *p* < 0.05), a contradictory yet not significant effect was observed for 0.1 µM AOH. Addition of PHTPP reduced abovementioned increase in IL6 expression (Figure 7A). The protein expression of IL6 evaluated with ELISA showed a remarkable increase in its expression after treatment with the higher dose of AOH (*** *p* < 0.001) as compared to control (Figure 7C). Blocking of ERβ with PHTPP decreased significantly expression of IL6 (* *p* < 0.05), but still the expression was elevated as compared to Cnt + PHTPP treatment (### *p* < 0.001). A contradictory effect was observed in the expression of interleukin 1β (*IL-1β*), where treatment with 10 µM AOH significantly (*** *p* < 0.001) decreased its expression and blockage of ERβ did not change that effect (## *p* < 0.01) as compared to Cnt + PHTPP (Figure 7B). The expression of IL-1β was not detectable at the protein level (data not showed).

## 3. Discussion

Toxins produced by *Alternaria*, including AOH are so-called “emerging mycotoxins”—a term defining the need for more evidence of their occurrence and toxicological properties. Although the human dietary exposure to AOH seems to be low, it exceeds the threshold of toxicological concern (TTC) [19] and there is still insufficient research concerning its mechanism and effects on human health [20]. This study evaluated for the first time the effect of AOH on the induction of oxidative stress in prostate normal epithelial cells via modulation of ERβ. The results presented here show that AOH induced oxidative stress in PNT1A cells is associated with DNA damage (Figure 3), cell cycle arrest in G2/M cell cycle phase (Figure 4) and that effect is partially caused by activation of ERβ confirming a previous statement that AOH possesses estrogenic effect in cells [9]. The doses used in the experiment were chosen on the basis of viability results and are consistent with the doses tested in other studies [10,15]. 

Although the observed cytotoxic effect of AOH in PNT1A cells (Figure 1) was only partially caused by activation of ERβ, it confirms the previous observation that AOH is more likely to affect ERβ then ERα [20]. The role of ERβ in prostate tissue is twofold—on one hand it is reported to act as tumor suppressor, lack of which results in carcinogenesis in mice [21], however on the other hand its expression is increasing in high tumor grades, indicating that ERβ suppresses proliferation of prostate cells, but stimulates its differentiation [22]. In this study, blocking of ERβ was not sufficient to completely reduce AOH-induced oxidative stress and DNA damage observed in PNT1A cells (Figure 2 and Figure 3), indicating that AOH-induced oxidative stress is mostly associated with its genotoxicity. The modulatory effect of estrogens in ROS induction in prostate cells was observed previously and seemed to be dependent on the ERα/ERβ ratio in cells [23]. AOH was previously reported to induce oxidative stress in human colon carcinoma cells in doses higher than 1 µM [24], whereas in human colon adenocarcinoma cells Caco-2 AOH induced oxidative stress in doses higher than 15 µM [25]. In our study, the concentration of 10 µM of AOH was sufficient to induce ROS generation in PNT1A cells (Figure 2). However, the induction of oxidative stress was still associated with activation of detoxifying enzymes (SOD1) as well as Nrf2 signaling pathway. AOH was reported to increase the translocation of Nrf2 to nuclei in HT29 cells [26]. In this study we observed that AOH significantly decreased expression of *NRF2* and modulated the expression of its responsive genes. A similar Nrf2 signaling pathway modulation effect of AOH was previously observed by us in normal mammary gland epithelial cells; however, in that study *NRF2* expression was not changed [27]. 

The toxic effect of AOH was previously mostly associated with its ability to act as topoisomerase inhibitor [6]. In this study we also observed that AOH induces DNA damage and cell cycle arrest in G2/M cell cycle phase (Figure 3 and Figure 4) was associated with modulation of the decreased expression of PARP1 and increased expression of *CCNB1* and *CDC2* main regulators of G2 cell cycle progression [28]. PARP1 modulation was reported in the study concerning the mycotoxin aflatoxin B1, in response to DNA damage [29], similarly to our study. The modulation of cell cycle progression was also associated with DNA damaging effect of AOH in RAW 264.7 cells [5], where, similarly to our study, G2/M cell cycle arrest was observed. 

ERβ was previously reported to participate in chronic inflammation in prostate via the NFκB HIF-1α signaling pathway [30]. In this study, as well as the induction of oxidative stress, we observed that AOH decreased the expression of *HIF1α* and that effect was reversed by locking ERβ with its selective inhibitor PHTPP. We also observed that AOH affects *RelA* expression. This result is in line with previous one indicating that AOH affects NFκB signaling pathway [18]. 

Extracellular matrix (ECM) molecules are considered as regulators of cells growth [31]. We observed that AOH significantly affected migration of PNT1A cells and that effect was associated with modulation of activity of MMP-2 and MMP-9 (Figure 6). Modulation of these components of ECM was previously reported as a factor in carcinogenesis and metastases, as well as age-related diseases [32]. The dual role of ERβ in prostate carcinogenesis was mentioned before: it seems that during early stages of prostate carcinogenesis ERβ acts as a tumor suppressor, but in more advanced disease it switches to a metastatic promoting role [33]. In this study we observed that lower concentration of AOH (0.1 µM) decreased migration of normal prostate epithelial cells, whereas co-incubation with an ERβ blocker caused a contradictory effect (Figure 6). The ECM role is also associated with inflammatory states [32]. IL-1β was associated with inflammation-induced invasion of prostate cancer cells [34]. AOH was previously reported to modulate the immune response in different cell lines [18,35]. IL-1β stimulated Caco-2 cells treated with AOH showed reduced IL-1β and IL6 expression [36]; decreased expression of IL6 and suppressed LPS-induced NFκB activation in THP-1 derived macrophages [18], whereas in RAW 264.7 mouse macrophages AOH increased IL6 expression [6]. The results of this study also showed that AOH modulates the expression of *IL6* (increase) and *IL-1β* (decrease) in normal prostate epithelial cells (Figure 7). IL6 was reported to modulate progression, differentiation, survival, and angiogenesis of PCa [36], moreover it was shown to mediate AR activation in benign and malignant prostate models [37]. Although ERβ is reported to suppress inflammation in prostate cells [38], in our study, blocking of ERβ decreased the expression of IL6, but still it seems that the immunomodulatory effect of AOH was sustained and visible in the increased *IL6* and decreased *Il-1β* expression. Previous research showed that AOH might act as immunomodulatory and xenoestrogenic agent. Bansal et al. observed that AOH-induced toxicity in derma cells is associated with inflammatory response to topical administration of AOH manifested by increased production of Cox-2 and PGE_2_ [39]. Another pro-inflammatory effect of AOH was reported in RAW264.7 mouse macrophages [6]. On the other hand, Kollarova et al. observed that AOH decreases LPS-induced inflammation in macrophages and proposed the molecular mechanism associated with modulation of NFΚB signaling pathway [18]. The mechanism of AOH-induced modulation of inflammatory response was evaluated by Favero et al. who suggested that structural similarity of AOH to cholesterol might provide a clue for understanding the biological effect triggered by AOH. Moreover, the authors suggested that AOH is more likely to affect signal transduction in cells, rather than its generation [40]. This fact seems to correspond to the results obtained by us, also to the estrogenic effect of AOH. This observations are also in line with previous one based on in silico and in vitro research, where metabolites of AOH triggered an estrogenic effect without direct binding to ERs in cells [41].

Taken together, AOH seems to act as immunomodulatory and estrogenic agent in cells and its biological effect might be dependent on the presence of other estrogenic stimuli, immunomodulatory agents as well as its metabolic modification in cells.

## 4. Conclusions

To the best of our knowledge, this is the first study which reports that AOH induces oxidative stress in normal prostate epithelial cells and that the effect is partially dependent on ERβ activation. The results of this study also confirm a previous reports indicating that AOH induces DNA damage, cell cycle arrest in G2/M cell cycle phase and acts as immunomodulatory agent affecting expression of IL6 and IL-1β. Due to the fact that hormonal imbalance and inflammation are crucial in both prostate benign and metastatic diseases, further research is needed to elucidate the role of AOH in prostate cells.

## 5. Materials and Methods

### 5.1. Cell Culture

PNT1A, normal human prostatic cell line, was supplied by the European Collection of Authenticated Cell Cultures (ECACC, Sigma-Aldrich, Saint Louis, MO, USA). Cells were cultured in an incubator under standard conditions (37 °C, 5% CO_2_, 95% humidity). RPMI-1640 with additives (10% FBS, 1% of sodium pyruvate, L-glutamine, HEPES and antibiotics) was used as a culture medium. For assays, an experimental medium without phenol red, antibiotics and FBS was used. 

### 5.2. Reagents & Treatments

AOH used in the study was derived from Sigma-Aldrich^®^. 2-Phenyl-3-(4-hydroxyphenyl)-5,7-bis (trifluoromethyl)-pyrazolo stock solution [1,5-α]pyrimidine (PHTPP) was derived from Santa Cruz Biotechnology (1 mM, Santa Cruz Biotechnology, Dallas, TX, USA). Both compounds were dissolved in DMSO and the final concentration was obtained by dissolution in experimental medium directly before treatment. The concentration of DMSO in final experimental medium was negligibly low (<0.01%) and did not affect the behavior of cells in all experiments. Based on viability test results, two doses of AOH were used (10 µM and 0.1 µM). Estradiol (E2) was used as positive control. As a control (Cnt), cells treated with the clear experimental medium were used. Combination of treatment: 10 µM AOH, 10 µM AOH + PHTPP, 0.1 µM AOH, 0.1 µM AOH + PHTPP, E2, E2 + PHTPP, and Cnt. 

### 5.3. Cell Viability and Morphology

The viability of cells was evaluated by AlamarBlue^®^ assay (Thermo Fisher Scientific Inc/Life Technologies, Saint Louis, MO, USA). Cells were seeded on 96-well plates (2 × 108 per well) and after one day, were treated with the experimental medium containing AOH in a concentration range of 0.001–100 µM for 24 and 48 h. After 20 h and after 44 h, respectively, 10 µL of AlamarBlue^®^ reagent was added and then plates were incubated in standard conditions for the next 4 h. Absorbance was measured at 570 nm and 600 nm in EL808IU BioTek microplate reader (BioTek Instruments, Inc., Winooski, VT, USA). Cell viability was expressed as percentage of Cnt cells. The cell viability assay was carried out in 6 replications.

### 5.4. Oxidative Stress, DNA Damage and Cell Cycle Distribution

All these flow cytometry assays were conducted with Muse^®^ Cell Analyzer in accordance with manufactuer’s recommendations (Luminex^®^, Austin, TX, USA). Cells were seeded on 6-well plates (ROS, Cell cycle) and 12-well plates (DNA damage) and then cultured in standard conditions until they reach 90% confluence. For oxidative stress the Muse^®^ Oxidative Stress Kit was used, for DNA damage Muse^®^ Multi-Color DNA Damage Kit and for cell cycle distribution Muse^®^ Cell Cycle Assay Kit. The experiments were conducted in triplicate.

### 5.5. Enzyme-Linked Immunosorbent Assay (ELISA) 

Enzyme-linked Immunosorbent Assays (ELISA) were conducted for IL-6 and IL-1β. The cells were seeded on 6-well plates and then treated with experimental medium as described above. After 24 h, the cells and experimental medium from the wells were harvested and frozen at −80 °C. Finally, the procedure was conducted according to the manufacturer’s instructions. The experiment was conducted in duplicate.

### 5.6. Scratch Assay

Wound scratch assay was performed to assess the ability of normal prostate cells to migrate after treatment with AOH. PNT1A cells were seeded on 6-well plates and allowed to reach 80–90% confluence. Then, cells were scratched in a cross shape with 200 µL sterile pipette tip, rinsed with DPBS (1×) and treated with previously prepared experimental medium. Cells were photographed immediately after addition of experimental medium (0 h) and after 24 h with Olympus DP20 camera (Olympus, magnitude 40×). Measurements of the difference between the wound area in 0 h and 24 h were used to determine migration. The wound area was measured in ImageJ software (https://imagej.nih.gov/ij/). The percentage of wound healing was calculated as shown below. The experiment was run in 3 replications.

x = (wound area (24 h))/(wound area (0 h))

The percentage of wound closure = (1 − x) × 100%

### 5.7. Gelatin Zymography

Gelatin zymography was performed to check whether the migration change after treatment with AOH was associated with activity of metalloproteinases (MMP-2 and MMP-9). For this purpose, cells were seeded on 6-well plates and allowed to reach 80–90% confluence. Next, cells were treated with AOH. Then, culture media were harvested and the concentration of the protein was determined using QubitR Protein Assay Kit (ThermoFisher Scientific, Inc, Waltham, MA, USA) according to the manufacturer’s instructions. 4.75 µg/µL of protein was used for the assay. Samples were electrophoresed (120 V, on ice) on 4% gelatin zymography gels, which were then incubated twice (30 min) in 2.5% Triton X-100 (Sigma-Aldrich, Saint Louis, MO, USA). The gels were then incubated in developing buffer (48 h, 37 °C). After the incubation time, the gels were stained with Coomassie brilliant blue (Sigma-Aldrich, Saint Louis, MO, USA), decolorized with decolorizing buffer (3(methanol):1(acetic acid):6(distilled water)) and preserved in glycine solution. The gels were photographed and ImageJ program/) was used to calculate the intensity of the bands. The experiment was carried out in 3 replications.

### 5.8. Real Time Quantitative Polymerase Chain Reaction (RT-qPCR)

Cells were seeded on Petri dishes (60 mm) and incubated in standard conditions until 90% confluence was achieved. Then, cells were treated with experimental medium as described in Section 5.2. After 24 h, total RNA was isolated using TRIzol reagent, according to the manufacturer’s instruction. BioDrop DUO was used to determine RNA purity and concentration (Biodrop, Cambridge, UK). 5 µg of RNA from each sample was used to synthesize cDNA by using ImProm RT-IITM reverse transcriptase (Promega, Madison, WI, USA). LightCycler 96 (Roche, Basel, Switzerland) was used to perform the RT-qPCR with 2 µL of cDNA. Primer-BLAST was used to design primers. To calibrate reaction, Human Reference RNA (Stratagene, San Diego, CA, USA) was used. Ribosomal protein S17 (*RPS17*), ribosomal protein P0 (*RPLP0*), and histone H3.3A (*H3F3A*) were used as a reference genes. Sequences of primers used in the study are presented in Table 5. Specificity of received product was confirmed during analysis of melting curves for each reactions. The ΔΔCt method was used to analyze the obtained data. The experiment was performed in duplicate with three independent replications.

### 5.9. Western Blot

Cells were seeded on Petri dishes (100 mm) and cultured to reach 90% confluence. Then, they were treated with experimental medium for 24 h. The protein isolation and western blots were conducted in accordance with previous study [16]. Primary antibodies used in the study were: anti-SOD1 (1:1000, Cell Signaling Technology, Leiden, WZ, The Netherlands, #71G8), anti-cleaved PARP1 (1:1000, Cell Signaling Technology, Leiden, WZ, The Netherlands, #D64E10), anti-GAPDH (1:2000, SantaCruz Biotechnology, Inc., Dallas, TX, USA, sc-59540). Novex^®^ AP Chromogenic Substrate (Thermo Fisher Scientific Inc, Waltham, MA, USA) was used to visualize bands. Quantification of bands intensity was measured with Image J software. The results are expressed as a relative expression normalized to control value (1.000).

### 5.10. Statistical Analysis

Data were analyzed in GraphPad Prism program (GraphPad Software version 5, La Jolla, CA, USA). Statistical significance was determined with One-Way ANOVA and post-hoc Bonferroni test. A *p* value lower than 0.05 indicates statistically significant results.

## Figures and Tables

**Table 1:** The relative expression of SOD1 obtained in Western blot. Quantification of bands intensity was measured with Image J software. The results are expressed as a relative expression normalized to control value (1.000). SOD1—superoxide dismutase 1, Cnt—control, AOH—alternariol, E2—estradiol.

	10 µM AOH	0.1 µM AOH	E2	Cnt	10 µM AOH + PHTPP	0.1 µM AOH + PHTPP	E2 + PHTPP	Cnt + PHTPP
SOD1	1.652	1.288	1.029	1.000	0.870	0.935	1.159	1.224

**Table 2:** The relative expression of genes obtained in RT-qPCR study. The results are expressed as mean value of at least three independent replications. One way ANOVA was used for statistical comparison. *p* < 0.05 was considered as statistically significant, ** *p* < 0.01, *** *p* < 0.001, as compared to 10 µM AOH. SOD1—superoxide dismutase 1, HIF-1α—hypoxia inducible factor 1α, RelA—nuclear factor NF-Kappa-B p65 subunit, Cnt—control, AOH—alternariol, E2—estradiol.

Gene	10 µM AOH	0.1 µM AOH	E2	Cnt	10 µM AOH + PHTPP	0.1 µM AOH + PHTPP	E2 + PHTPP	Cnt + PHTPP
*SOD1*	1.97	2.45	2.25	2.37	2.04	2.98	2.51	2.58
*HIF-1* *α*	0.74	0.94	0.80	0.86	1.07 **	0.99	0.84	0.83
*RelA*	13.91	21.08	16.61	18.08	27.61 ***	29.09	22.83	32.63

**Table 3:** The relative expression of cleaved- PARP1 obtained in Western blot. Quantification of bands intensity was measured with Image J software. The results are expressed as a relative expression normalized to control value (1.000). PARP1-Poly [ADP-ribose] polymerase 1, Cnt—control, AOH—alternariol, E2—estradiol.

	10 µM AOH	0.1 µM AOH	E2	Cnt	10 µM AOH + PHTPP	0.1 µM AOH + PHTPP	E2 + PHTPP	Cnt + PHTPP
Cleaved-PARP1	0.616	0.732	0.625	1.000	0.767	0.868	0.847	0.846

**Table 4:** The activity of MMP-2 and MMP-9 obtained during zymography assay. The results are expressed as mean % of the control, of three replicates. One way ANOVA was used for statistical comparison. *p* < 0.05 was considered as statistically significant. MMP-2 metalloproteinase 2, MMP-9—metalloproteinase 9, AOH—alternariol, Cnt—control, E2—estradiol.

Gene	10 µM AOH	0.1 µM AOH	E2	Cnt	10 µM AOH + PHTPP	0.1 µM AOH + PHTPP	E2 + PHTPP	Cnt + PHTPP
*MMP-2*	81.86	69.76	86.22	100.0	79.47	84.82	93.27	91.63
*MMP-9*	91.16	94.22	100.9	100.0	98.74	110.6	98.44	96.52

**Table 5:** Sequences of primers used in RTqPCR.

Gene	Sequence (5′-3′)	Product Size [bp]
*CDC2*	For TTTTCAGAGCTTTGGGCACT Rev AGGCTTCCTGGTTTCCATTT	100
*CCNB1*	For ACCTATGCTGGTGCCAGTG Rev GGCTTGGAGAGCAGTA	128
*GCLM*	For TGTGCAACTCCAAGGACTGA Rev ACAGCGAGGAGCTTCATGAT	247
*HIF-1α*	For TTACTCATCCATGTGACCATGA Rev AGTTCTTCCTCGGCTAGTTAG	140
*HMOX1*	For CAGCTCCTGCAACTCCTCAAA Rev TTCTTCACCTTCCCCAACATTG	165
*H3F3A*	For AGGACTTTAAAAGATCTGCGCTTCCAGAG Rev ACCAGATAGGCCTCACTTGCCTCCTGC	74
*IL-1β*	For GGCAATGAGGATGACTTGTT Rev TGCTGTAGTGGTGGTCGGA	127
*IL6*	For GGATGCTTCCAATCTGGATTCA Rev TCTGGCTTGTTCCTCACTACT	126
*NQO1*	For CCAGGATTTGAATTCGGGCG Rev AGGACCCTTCCGGAGTAAGA	212
*NRF2*	For GTCACATCGAGAGCCCAGTC Rev ACCATGGTAGTCTCAACCAGC	193
*PARP1*	For TCTTCAAGAGCGATGCCTATT Rev TGAGGTAAGAGATTTCTCGGAA	129
*RelA*	For GCACAGATACCACCAAGACC Rev TCAGCCTCATAGAAGCCATC	157
*RPLP0*	For ACGGATTACACCTTCCCACTTGCTAAAAGGTC Rev AGCCACAAAGGCAGATGGATCAGCCAAG	69
*RPS17*	For AAGCGCGTGTGCGAGGAGATCG Rev TCGCTTCATCAGATGCGTGACATAACCTG	87
*SOD1*	For GCGTGGCCTAGCGAGTTAT Rev. ACACCTTCACTGGTCCATTACT	114

## Data Availability

Data are available upon request.
